# Interspecific Infanticide and Infant-Directed Aggression by Spider Monkeys (Ateles hybridus) in a Fragmented Forest in Colombia

**DOI:** 10.1002/ajp.22052

**Published:** 2012-07-05

**Authors:** Rebecca Rimbach, Alejandra Pardo-Martinez, Andres Montes-Rojas, Anthony Di Fiore, Andres Link

**Affiliations:** 1 Fundación Proyecto PrimatesBogota, Colombia; 2 Behavioral Ecology and Sociobiology Unit, German Primate CenterGöttingen, Germany; 3 Departamento de Biologia, Universidad Nacional de ColombiaBogotá, Colombia; 4 Departamento de Biologia, Universidad del TolimaIbagué, Colombia; 5 Department of Anthropology, University of Texas at AustinAustin, Texas; 6 Laboratorio de Ecología de Bosques Tropicales y Primatología, Departamento de Ciencias Biológicas, Universidad de Los AndesBogotá, Colombia

**Keywords:** interspecific aggression, infanticide, *Ateles*, resource competition, pathological behaviors

## Abstract

Interspecific aggression amongst nonhuman primates is rarely observed and has been mostly related to scenarios of resource competition. Interspecific infanticide is even rarer, and both the ultimate and proximate socio-ecological factors explaining this behavior are still unclear. We report two cases of interspecific infanticide and five cases of interspecific infant-directed aggression occurring in a well-habituated primate community living in a fragmented landscape in Colombia. All cases were initiated by male brown spider monkeys (*Ateles hybridus*) and were directed toward infants of either red howler monkeys (*Alouatta seniculus*: *n* = 6 cases) or white-fronted capuchins (*Cebus albifrons*: *n* = 1 case). One individual, a subadult spider monkey male, was involved in all but one case of interspecific infanticide or aggression. Other adult spider monkeys participated in interspecific aggression that did not escalate into potentially lethal encounters. We suggest that competition for food resources and space in a primate community living in high population densities and restricted to a forest fragment of ca. 65 ha might partly be driving the observed patterns of interspecific aggression. On the other hand, the fact that all but one case of interspecific infanticide and aggression involved the only subadult male spider monkey suggests this behavior might either be pathological or constitute a particular case of redirected aggression. Even if the underlying principles behind interspecific aggression and infanticide are poorly understood, they represent an important factor influencing the demographic trends of the primate community at this study site. Am. J. Primatol. 74:990–997, 2012. © 2012 Wiley Periodicals, Inc.

## INTRODUCTION

Interspecific high-intensity aggression—or aggression seemingly aimed at wounding or killing an individual of another species—is a rarely observed behavior in mammals [Cassini, 1998; Cotter et al., 2012; Peiman & Robinson, 2010] except in the context of predator–prey interactions. Among primates, the most common form of interspecific lethal aggression is also observed in those few species that prey upon other sympatric primates [e.g., chimpanzees: Stanford et al., 1994; Stanford, 2002; Watts & Mitani, 2002]. Although rarely observed, some cases of interspecific aggression have been also reported among primate species outside of predator–prey interactions, mainly in the context of interspecific resource competition at actual feeding sites [Heymann, 1990; Stevenson et al., 2000]. For example, in Costa Rica, aggression between white-faced capuchins (*Cebus capucinus*) and black-handed spider monkeys (*Ateles geoffroyi*) can arise at heavily contested food resource sites [Rose et al., 2003]. At the same study site, Rose et al. [2003] also observed aggression between white-faced capuchins (*C. capucinus*) and mantled howler monkeys (*Alouatta palliata*) that was not obviously associated with resource competition.

Infanticide—the act of killing a dependent offspring usually committed by a conspecific—is a fairly common behavior in a wide variety of taxonomic groups, including beetles [Trumbo, 1990], fish [Kondoh & Okuda, 2002], birds [Weisheit & Creighton, 1989], rats [Boice, 1972], lions [Packer & Pusey, 1983], nonhuman primates [Agoramoorthy & Rudran, 1995; Butynski, 1982; Newton, 1988] and humans [Hausfater, 1984; Riches, 1974]. Although many of the hypotheses forwarded to explain the occurrence of infanticide are related to male reproductive strategies or intersexual conflict, some individuals commit infanticide without any obvious prospect of a personal fitness gain. Several adaptive hypotheses have been proposed to explain the potential benefits of infanticide, particularly to males, who are responsible for most cases of infanticide reported in the primate literature: [1] the use of an infant as a food resource, [2] the elimination of handicapped or ill-timed offspring by a parent which could lead to improved lifetime reproductive success, [3] the elimination of a potential future competitor for resources, and, specifically for males, [4] the elimination of the offspring of male rivals and the hastening of a female's return to normal cycling following loss of her infant [the “sexual selection” hypothesis: Hrdy, 1979; van Schaik, 2000].

Additionally, the “social pathology” hypothesis for infanticide has been proposed to explain certain cases of infanticide as an apparently maladaptive social behavior or an epiphenomenon associated with aggression generally, which conveys no apparent benefits to committing infanticide [Curtin, 1977; Curtin & Dolhinow, 1978; Hrdy, 1979; Sussman et al.*,* 1995]. Under some circumstances, wild animals might be exposed to high stress levels (e.g., in captivity or in recently disturbed areas) and might display unnatural and destructive behaviors [Dolhinow, 1977]. Under this scenario, infanticide should be unpredictable and often associated with the vulnerability of young animals [Curtin & Dolhinow, 1978].

Interspecific infanticide is an extremely rare behavior amongst mammals [see Cassini, 1998]. One of the few examples of interspecific infanticide among mammals has been documented for South American sea lions (*Otaria flavescens*), which occasionally kill infants of South American fur seals (*Arctocephalus australis*) at islands where both species reproduce in sympatry [Cassini, 1998]. Subadults are the main perpetrators of such infanticide, and it has been hypothesized that these cases of infant killing are in fact a byproduct of sexual conflict among these individuals [Cassini, 1998]. Interspecific infanticide has also been reported in tropical house wrens (*Troglodytes aedon*) by rufous-and-white wrens (*Thryothorus rufalbus*) [Freed, 1987]. Cases of infanticide between these two wren species were almost certainly based on food resource competition, since nestlings were killed only during a pronounced food shortage. Barn owls (*Tyto alba*) have been reported to increase their reproductive success by killing the broods of tawny owls (*Strix aluco*) in times of breeding place shortage (Mátics et al., 2008). The underlying causes of interspecific infanticide are still poorly understood, as the key adaptive hypothesis proposed to explain conspecific infanticide [e.g., the “sexual selection” hypothesis: Hrdy, 1979; van Schaik, 2000] cannot be applied to cases of interspecific infant killing.

Here, we report several cases of interspecific infanticide and infant-directed aggression that we observed in a well-habituated primate community living in a fragmented landscape in Colombia. We describe all events where at least one member of a group of brown spider monkeys (*Ateles hybridus*) initiated high intensity or lethal aggression toward infants of other sympatric primates.

## METHODS

### Study Site

The study took place in a forest fragment located within the private cattle ranch “Hacienda San Juan del Carare” (06°43′N, 74°09′W), where studies on the behavioral ecology of brown spider monkeys (*A. hybridus*), red howler monkeys (*Alouatta seniculus*) and white-fronted capuchin monkeys (*Cebus albifrons*) have been conducted since 2007. The study site is located near the Magdalena River between the eastern and central cordilleras of the Andes of Colombia. The study site is a thin forest patch of roughly 65 ha of seasonally flooded tropical rainforest. The forest fragment runs along the western bank of the San Juan River and is surrounded on its western border by natural savannas and wetlands and on its eastern border by the San Juan River. In 2007, prior to the onset of our long-term work at the site, the forest was isolated from a larger expanse of forest by the clearing of trees and conversion to pastures of land to the north and south of the fragment. Although dispersal to other nearby fragments is feasible for *C. albifrons*, which can use low vegetation to cross between fragments, it is likely to be severely constrained for both *Alouatta seniculus* and *A. hybridus*. All three diurnal species in the primate community at the study site live at high population densities: 42.8 ind./km^2^ of *A. hybridus*, 98.8 ind./km^2^ of *Alouatta seniculus,* and 130.3 ind./km^2^ of *C. albifrons* [Link et al., 2010].

### Data Collection

Data were collected on one of the two groups of brown spider monkeys that live within the forest fragment at San Juan. The study group consisted of three adult males (one of whom disappeared partway through the study, in June 2011), five adult females with offspring, and one subadult male; all group members were well habituated and individually recognized. We conducted behavioral follows from dusk to dawn on focal individuals from this group and used all day focal animal sampling [Altmann, 1974] to collect behavioral, ranging, and foraging data. These data were complemented with ad libitum data on conspicuous behaviors such as aggression and intergroup encounters. We also conducted behavioral follows on six groups of howler monkeys and two groups of white-faced capuchins at the site. During the study period (January 2010 to December 2011), all three sympatric taxa were followed by at least one researcher, obtaining roughly 3000, 1500, and 800 hr of behavioral follows on groups of *A. hybridu*s, *Alouatta seniculus,* and *C. albifrons*, respectively. All cases of interspecific infanticide were recorded during behavioral follows, and, given the conspicuous nature of intergroup aggression, we were able to record in detail all such events. This research adhered to the American Society of Primatologists principles for the ethical treatment of primates, complied with protocols approved by IACUC at New York University and adhered to the legal requirements of the Colombian legislation.

## RESULTS

Over the course of the study, we observed two cases of interspecific infanticide and five cases of interspecific infant-directed aggression which not led to the death of the infant. These are summarized in Table 1 and described in more detail below.

**TABLE 1:** Observed Cases of Interspecific Infanticide and Infant-Directed Aggression by Spider Monkeys

Case	Type	Date	Target species	Age-sex class of aggressor(s)	Age-sex class of victim
1	Infanticide	August 16,2010	*Alouatta*	**One subadult male**	**One infant male**
			*Seniculus*		
2	Infant-directed aggression	August 18, 2010	*Alouatta*	**One subadult male**	**One infant (unknown sex)**
			*Seniculus*		
3	Infant-directed aggression	January 17, 2011	*Alouatta*	**One subadult male**	**One infant (unknown sex)**
			*Seniculus*	One adult male	One adult male
4	Infant-directed aggression	June 21, 2011	*Alouatta*	**One subadult male**	**One infant male**
			*Seniculus*	Two adult males	
				Five adult females	
5	Infanticide	June 30, June 2011	*Alouatta*	**One subadult male**	**One infant (unknown sex)**
			*Seniculus*		
6	Infant-directed aggression	July 05,2011	*Alouatta*	**One subadult male**	**One infant (unknown sex)**
			*Seniculus*		
7	Infant-directed aggression	August 20, 2011	*Cebus albifrons*	**Two adult males**	**One infant (unknown sex)**

Note: Bold type indicates the main initiator and main target of high intensity aggression.

### Observations

#### Case 1 (Infanticide)

**Fig. 1:**
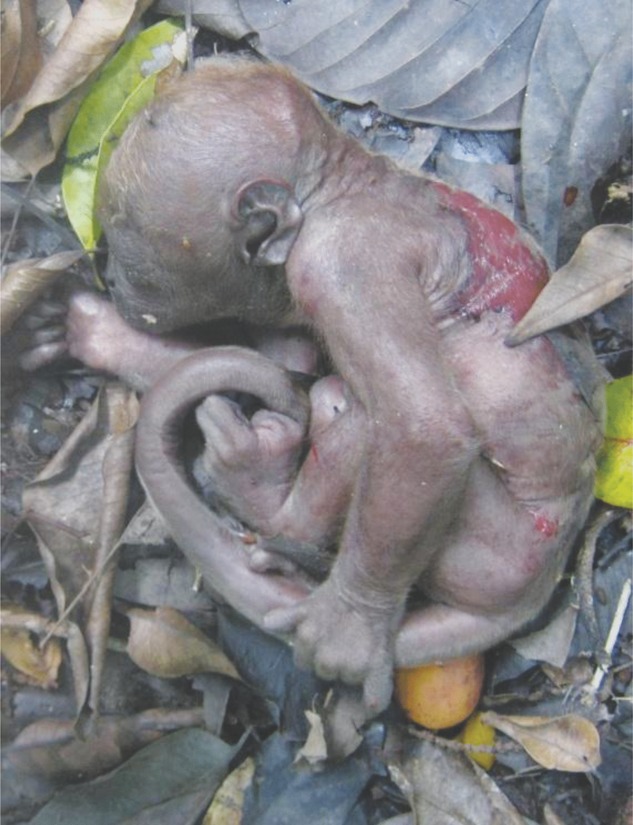
Infant male howler monkey (*A. seniculus*), after the second fall described in Case 1, while still alive. (Photograph by R. Rimbach).

On August 16, 2010, we followed a subgroup of spider monkeys, which contained the only subadult spider monkey male of the group as the focal animal. The subadult male was traveling apart from the rest of the subgroup, and at 08:50, we heard loud “growling” vocalizations (which are typically emitted during aggressive interactions). We approached the location of those vocalizations, and at 08:54, we found a newborn male red howler monkey (with a piece of umbilical cord still attached) on the forest floor. We kept at a distance from the infant, and at 09:04, an adult female howler monkey (presumably the newborn's mother) descended to the forest floor and retrieved the infant. As she climbed back onto the tree, we observed the subadult male spider monkey directing aggression toward the howler monkey female. He grabbed at the infant, which then fell again to the forest floor (Fig. 1). At 09:39, shortly after the subadult male spider monkey left the area, the female picked up the injured infant again. After the second fall, the infant was not able to hold onto the female, and the female was not able to support him while climbing in the tree. He fell again to the ground at 09:40. During the course of the day, he fell four more times to the forest floor and died during the late afternoon that same day. Although the other members of the howler monkey group (an adult male, another adult female, and a juvenile male) were present during the entire event, neither did they emit alarm calls nor interfere with the subadult spider monkey's aggression. The dead infant's body had several small round wounds (arranged in a half circle) on his back, which we infer were caused by the teeth of subadult spider monkey.

#### Case 2 (Infant-directed aggression)

On August 18, 2010 (two days after case 1), we were again following a subgroup of spider monkeys containing the same subadult male. He was moving at the rear of the subgroup. At 17:06, we again heard “growling” vocalizations and immediately noticed another newborn red howler monkey on the forest floor. One of the two females of the howler monkey group (presumably the mother) retrieved the infant (of unknown sex) from the ground. The subadult spider monkey male immediately approached the female, grabbed the infant by its tail, and chased the female howler monkey for ca. 4 min. before leaving the area. The female did not alarm call and again none of the other group members interfered with the aggression nor vocalized. This infant howler monkey survived the incident with no apparent injuries and subsequently was carried by a female of the group.

#### Case 3 (Infant-directed aggression)

On January 17, 2011, we followed a subgroup of spider monkeys that consisted of two adult females, their offspring, an adult male, and the same subadult male. At 09:44, the subadult male initiated aggression toward a howler monkey group, and the other adult spider monkey male present in the subgroup joined in. The subadult male chased a female howler monkey carrying an infant of about one month old. He grabbed the infant from its mother, threw it to the ground, and then continued chasing the female. The infant stayed on the ground for a few seconds and then climbed up into a shrub, where the female then retrieved it. She rapidly moved away from the subadult spider monkey, while he stayed several meters behind staring at her. Meanwhile, the adult male spider monkey attacked an adult howler monkey male and chased him for 20 meters until the howler monkey turned and confronted him. The adult male spider monkey stopped the attack and the adult male howler monkey left the tree. During the aggression, the other howler monkey group members neither interfered nor vocalized.

#### Case 4 (Infant-directed aggression)

On June 21, 2011, we followed the entire group of spider monkeys, which consisted at the time of five adult females, their offspring, two adult males, and the subadult male. At 11:00, the subadult male initiated an aggression toward a group of howler monkeys, shaking branches and chasing them, and he was joined by the other adult spider monkeys. The subadult male attempted to take an infant howler monkey off the back of its mother. The other howler monkey group members retreated without defending either the female or the infant. One minute later, the subadult male spider monkey pulled the infant by its tail while at the same time its mother tried to hold him by its arms. At 11:04, the infant fell to the ground from ca. 20 meters up in the canopy, and the female was not able to descend to the ground to retrieve the infant because the subadult male continued his aggression toward her. She was forced to leave the tree, and soon after the subadult male spider monkey descended to the ground three times to observe the infant. The male infant was about two months old. He had a deep wound on one leg and about a third of its tail was ripped off. The infant vocalized while laying on the ground, and 2 hr later the female howler monkey appeared again. She tried to recover the infant but the subadult spider monkey again directed aggression toward her. At 14:47, the female finally descended to the ground and successfully retrieved the infant. She went up into the trees and left the area rapidly. We could not confirm whether or not the infant survived.

#### Case 5 (Infanticide)

**Fig. 2:**
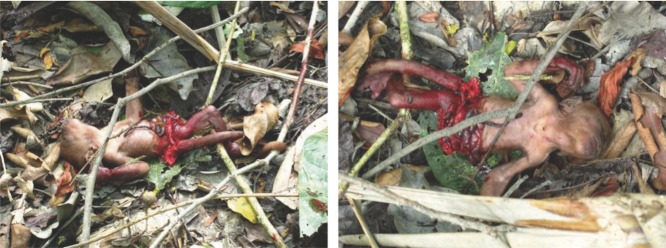
Infant howler monkey after being attacked by a subadult spider monkey described in Case 5. (Photographs by A. Pardo Martinez).

On June 30, 2011, the same subadult spider monkey male was resting with three adult females, their offspring, and the two adult males. At 12:30, the subadult male and another adult male left the other group members and approached a group of howler monkeys, which immediately started alarm calling and began to leave the tree. The subadult male then attacked a female howler monkey with an infant on her back. He chased the female, grabbed the infant, and pulled it by its tail. At 12:45, the subadult male bit the infant's body inflicting a large open wound on its abdomen. The attack lasted a few seconds, and the infant fell to the ground while the female escaped. The subadult male remained at approximately 1.5 m watching the infant for several minutes. The infant was still alive, although it had serious wounds in the abdomen that left its internal organs exposed (Fig. 2). The subadult male descended to the ground, observed the infant, and then returned to the tree and kept watching it for 5 min more. At 12:52, he left the area. The other howler monkeys did not interfere with the aggressor, and the female did not return to check on her infant. We did not retrieve the infant's body when we left because it was still alive and kept vocalizing. But due to the severity of its wounds, it is highly probable that the infant died.

#### Case 6 (Infant-directed aggression)

On July 5, 2011, we followed a female spider monkey, her offspring, and the subadult male. At 13:02, the subadult male moved toward group of howler monkeys that was resting about 20 m away. He emitted several growling vocalizations and then began directing aggression toward an adult female howler monkey that had an infant on her back. The female rapidly jumped into a small tree close to the forest ground, and the subadult male followed her. When the female was approximately 2 m above the ground, the subadult male stopped chasing her but vocalized and displayed by shaking branches at her. He remained staring at her for about 2 min before leaving them at 13:05. The other howler monkey group members emitted alarm calls during the aggression.

#### Case 7 (Infant-directed aggression)

On August 20, 2011, we followed a subgroup of spider monkeys that consisted of five adult females, their offspring, and two adult males. At 09:05, the adult males were foraging far from the rest of the group close to a group of white-fronted capuchin monkeys that was also foraging. At 09:11, a female capuchin monkey with an infant on her back passed in front of the spider monkey males, and they began directing aggression toward her. The female capuchin tried to escape from the males, but they reached her and took off her infant. The female struggled with them and the infant fell from around 10 m to the ground. It stayed there for a few seconds and then went up a small tree. The female escaped from the males by descending to the ground and hiding in the shrubs where they could not follow her. The aggression ended at 09:14, and the infant survived the incident without injuries.

## DISCUSSION

Interspecific aggression and infanticide is a rare and poorly understood behavior amongst primates. Nonetheless, although other behaviors such as conspecific infanticide and predation are also rarely observed in most primatological field studies, they still feature prominently in contemporary socioecological models concerning the evolution of primate sociality [Isbell 1994; van Schaik, 1996]. During our two-year study, we observed seven cases of interspecific conflict between spider monkeys and two other sympatric primate species (the only other diurnal species found in the community), at least two of which escalated into lethal aggression. Interestingly, most populations of spider monkeys live sympatrically with howler monkeys and capuchin monkeys, but even during long-term studies conducted on *Ateles* at other sites, this type of behavior has not been reported previously, although spider monkey males have been observed to cooperatively chase and aggressively interact with other mammal species like coatis (*Nasua nasua*) and sloths (*Bradypus variegatus*) (Link et al., personal observation). Furthermore, at most study sites, juvenile spider monkeys have been observed playing for extended periods with other juvenile primates, including howler monkeys and woolly monkeys [Link, Di Fiore, personal observations]. In all cases of interspecific infanticide or infant-directed aggression reported in this study, male spider monkeys exclusively directed the aggression to females that carried dependent offspring and attempted to inflict injuries on their infants.

Interestingly, in all but one of the cases we describe, a single individual (the only subadult male spider monkey) was involved in and acted as the initiator and principal perpetrator of aggressive interspecific interactions. It is thus possible that this subadult male spider monkey is a particularly aggressive individual (or at least was particularly aggressive during this specific observation period), or that his behavior is somehow “pathological,” or both. Alternatively, his behavior could be a result, in part, of the fact that he was reaching adulthood throughout this study, a period when male spider monkeys are exposed to high levels of stress because of intraspecific aggression targeted at them from older males, which can sometimes even escalate to lethal aggression [Campbell, 2006; Valero et al., 2006]. In fact, most of the aggression we observed within the spider monkey group was directed toward this particular subadult male [Link et al., unpublished data], and thus, his pervasive participation in infanticide-related behaviors could conceivably reflect “redirected aggression”. For many primates, it has been found that levels of aggression of males rise during sexual maturation [Anestis, 2006; Kramer et al., 1982; Pereira & Altmann, 1985]. Cassini [1998] reports that only subadult sea lion males (*O. flavescens*)—and never adults—attack and sometimes even kill seal pups (*A. australis*). Still, the fact that the subadult male spider monkey was repeatedly joined in his aggressive interspecific interactions by other adult individuals (mostly, but not exclusively, adult males) suggests that such aggression at San Juan might constitute more than merely individual pathological behavior.

Out of the four possible adaptive hypotheses positing fitness gains for the perpetrators of infanticide [Hrdy, 1979], those related to improving reproductive opportunities for infanticidal males after killing infants of females of another taxon can be ruled out. The elimination of potential competitors for food resources remains as a potential explanation for the reported cases. Under conditions of high population density, species that overlap in their ecological niches may experience increased resource competition; thus, during these times infanticide could conceivably be used as a strategy to eliminate potential future competitors for resources or space. Accordingly, it has been suggested that resource competition is the most plausible explanation for male infanticide in male bank voles (*Myodes glareolus*). At high population densities, aggressiveness of male bank voles toward pups of their own species increases [Korpela et al., 2010]. Moreover, resource competition also consistently affects interspecific aggressive interactions and nest predation between bank voles and common shrews (*Sorex araneus*) [Liesenjohann et al., 2011]. Among primates, Rose et al., [2003] observed multiple cases of aggression directed by white-faced capuchins (*C. capucinus*) toward mantled howler monkeys (*A. palliata*) as well as cases of reciprocal aggression (mostly chases and threats) between capuchins and black-handed spider monkeys (*A. geoffroyi*) at the same site. Most of the confrontations between the latter two primate species arose over heavily contested resources, suggesting resource competition might be driving this interspecific aggression.

The members of the primate community we studied at San Juan live at very high density [Link et al., 2010] in a small forest fragment of only 65 ha. Under these conditions, resource competition for food and space may indeed be a possible explanation for the observed cases of interspecific aggression and infanticide we report here. Although none of the cases of infant-directed aggressions we observed took place during contest competition for feeding resources, the dietary overlap between spider monkeys and howler monkeys is considerably high in this forest fragment. Depending on the season, the diet of spider monkeys can contain a very high percentage of new leaves 26–57% [Aldana-Saavedra, 2009]—considerably higher than that reported at other study sites where leaves comprise a much smaller fraction of the diet [e.g., 8.3%: Nunes, 1998, 9%: Dew, 2001; 12%: Russo et al., 2005; 17.2%: Campbell, 2000, reviewed in Di Fiore et al., 2011]. At the same time, howler monkeys seasonally consume a high percentage of ripe fruits at the study site [45–75%: San Juan, Aldana-Saavedra, 2009]. In contrast, there is much less dietary overlap between capuchins and spider monkeys because capuchins have a very flexible diet and feed on fruits, insects, small vertebrates, and mammals.

Even though interspecific infanticide has been seldom documented in primate studies, it seems that it could be an important factor influencing the population dynamics of howler monkeys at San Juan. If it turns out that interspecific infanticide in this situation is largely explained by the “pathological” behavior of a single individual, then the phenomenon may only be relevant for this *particular* community and only during the next few years. On the other hand, if interspecific infanticide represents a more general phenomenon influenced by high population densities and resource competition in recently fragmented forests, then we might predict that similar behaviors will increasingly be observed in populations of primates that are being isolated in forest fragments at high population density as habitat fragmentation and isolation due to human activity increases worldwide.
